# Supplementary material for: The gender gap in aversion to COVID-19 exposure: Evidence from professional tennis
Source: PLoS One. 2021 Mar 25;16(3):e0249045. doi: 10.1371/journal.pone.0249045 (PMC7993799; doi:10.1371/journal.pone.0249045)
Supplement: S1 Table — (DOCX) [file pone.0249045.s001.docx]

**S1 Table. Players who withdrew from the 2020 US Open because of COVID-19**

| **Ranking** | **Name** | **Country** | **Age** | **Ranking points** | **Tournaments played in the last year** | **Gender** |
| --- | --- | --- | --- | --- | --- | --- |
| 1 | Ashleigh Barty | AUS | 24 | 8717 | 17 | Female |
| 2 | Rafael Nadal | ESP | 34 | 9850 | 18 | Male |
| 2 | Simona Halep | ROU | 29 | 6356 | 17 | Female |
| 5 | Elina Svitolina | UKR | 26 | 4580 | 24 | Female |
| 6 | Bianca Andreescu | CAN | 20 | 4555 | 10 | Female |
| 7 | Kiki Bertens | NED | 29 | 4335 | 25 | Female |
| 9 | Gael Monfils | FRA | 33 | 2860 | 22 | Male |
| 10 | Belinda Bencic | SUI | 23 | 4010 | 25 | Female |
| 15 | Stan Wawrinka | SUI | 35 | 2185 | 20 | Male |
| 29 | Qiang Wang | CHN | 28 | 1706 | 23 | Female |
| 30 | Anastasia Pavlyuchenkova | RUS | 29 | 1540 | 22 | Female |
| 31 | Barbora Strycova | CZE | 34 | 1530 | 21 | Female |
| 32 | Svetlana Kuznetsova | RUS | 35 | 1527 | 17 | Female |
| 34 | Saisai Zheng | CHN | 26 | 1510 | 24 | Female |
| 40 | Nick Kyrgios | AUS | 25 | 1170 | 18 | Male |
| 38 | Julia Goerges | GER | 32 | 1423 | 21 | Female |
| 42 | Jelena Ostapenko | LAT | 23 | 1360 | 24 | Female |
| 47 | Polona Hercog | SLO | 29 | 1205 | 23 | Female |
| 49 | Jo-Wilfried Tsonga | FRA | 35 | 1005 | 22 | Male |
| 52 | Fernando Verdasco | ESP | 36 | 945 | 25 | Male |
| 56 | Su-Wei Hsieh | TPE | 34 | 1035 | 23 | Female |
| 73 | Tamara Zidansek | SLO | 23 | 840 | 26 | Female |
| 76 | Lin Zhu | CHN | 26 | 830 | 30 | Female |
| 81 | Yafan Wang | CHN | 26 | 795 | 26 | Female |
| 89 | Andrea Petkovic | GER | 33 | 750 | 23 | Female |

Source: Own elaboration based on data web scraped from ATP, WTA, and US Open websites.
