# Supplementary material for: The gender gap in aversion to COVID-19 exposure: Evidence from professional tennis
Source: PLoS One. 2021 Mar 25;16(3):e0249045. doi: 10.1371/journal.pone.0249045 (PMC7993799; doi:10.1371/journal.pone.0249045)
Supplement: S2 Table — (DOCX) [file pone.0249045.s002.docx]

**S2 Table. Players who withdrew from the 2020 US Open for other reasons**

| **Ranking** | **Name** | **Country** | **Age** | **Ranking points** | **Tournaments played in the last year** | **Gender** | **Withdrawal reason** |
| --- | --- | --- | --- | --- | --- | --- | --- |
| 4 | Roger Federer | SUI | 39 | 6630 | 16 | Male | Injury |
| 11 | Fabio Fognini | ITA | 33 | 2400 | 24 | Male | Injury |
| 22 | Benoit Paire | FRA | 31 | 1738 | 32 | Male | Diagnosed with COVID |
| 31 | Kei Nishikori | JPN | 30 | 1345 | 13 | Male | Diagnosed with COVID |
| 45 | Fiona Ferro | FRA | 23 | 1267 | 26 | Female | Injury |
| 58 | Lucas Pouille | FRA | 26 | 880 | 22 | Male | Injury |
| 68 | Pierre-Hugues Herbert | FRA | 29 | 765 | 28 | Male | Parenting |
| 69 | Carla Suárez Navarro | ESP | 32 | 881 | 19 | Female | Illness |
| 85 | Anastasia Potapova | RUS | 19 | 759 | 27 | Female | Injury |
| 87 | Ana Bogdan | ROU | 28 | 755 | 26 | Female | Injury |
| 98 | Samantha Stosur | AUS | 36 | 667 | 22 | Female | Parenting |

Source: Own elaboration based on data web scraped from ATP, WTA, and US Open websites.
