# Supplementary material for: The gender gap in aversion to COVID-19 exposure: Evidence from professional tennis
Source: PLoS One. 2021 Mar 25;16(3):e0249045. doi: 10.1371/journal.pone.0249045 (PMC7993799; doi:10.1371/journal.pone.0249045)
Supplement: Attachment — Submitted filename: Response to reviewer 1.docx [file pone.0249045.s004.docx]

Thank you for your comments and suggestions which we have accounted for in the revised draft. We have elaborated the implications of our findings in the “conclusions” section. We have also reorganized the manuscript and edited the presentation of our results and findings in order to facilitate readability. Below we respond to each of your main comments.

*• Lines 50-56: As this paragraph involves discussion of the results, it seems it should be moved out of the introduction of the manuscript*

We would prefer to retain this paragraph as it helps us to show our contribution.

*• Lines 124-125: Please provide further detail for readers on the Shapley decomposition method*

We have explained the method in more detail.

*• Lines 160-163; 165-168: Please clarify how the percentage of explained variance is obtained or calculated. Can this be discerned from Figure 2? In the figure 2 caption, please provide greater detail and clarify what the x-axis represents.*

We have replaced figure 2 with a table (Table 3) that includes the contributions of particular factors to overall variance, as well as their shares in total explained variance which were discussed in the text but not shown explicitly in figure 2. In the current draft, all data cited in the text are shown in Table 3.

*• Discussion: The last two paragraphs in the discussion section seem like they should be swapped. Ending the manuscript by discussing the limitations minimizes the impact of the work so I would suggest addressing limitations and then finishing with the concluding paragraph.*

We have swapped these paragraphs and elaborated the (now final) paragraph on broader implications of our findings.

*• Line 51: “Higher-ranked”
• Line 85: Word missing? “…study (to) the…”
• Line 103 and throughout: I believe text concerning the methodology should be in past tense (i.e. “…we estimated logistic…)*

*• Line 133: Please write out what “pp” indicates before using the abbreviation
• Lines 170-172: Seems like a word or two is missing in this sentence as it is unclear what is being discussed (“…while the contribution of country-level preferences…”)*

We have edited the text accordingly.

*• Line 123: Please briefly clarify for readers why probit and linear probability models were estimated in addition to a logit model.*

We have added an explanation: we do it in order to test whether our findings are robust to changes in estimation method. We find that our results do not depend on choice of the estimation method.

*• Lines 186-187: Please discuss further the result that 15% of the withdrawal probability explained can be attributed to gender. This seems low, especially given the disparity in withdrawals between males and females. What could explain the other 85%? Is the low percentage explained a limitation and does it hinder the ability to draw strong conclusions about gender differences from these data?*

We have elaborated this discussion. Overall, our models explain 20-30% of the overall variance in the total sample and various subsamples. We think it’s a decent share considering that the decision about participation may be affected by individual factors and past experiences, but the data allows to control only for sex, age, and ranking, and country-level variables (cultural preferences, level of development).

*• Do females typically have longer professional careers than males? If so, they may feel they are better able to accommodate skipping a tournament. Just a thought.*

In general, women reach their highest career level earlier than men, which is consistent with their more precocious biological development. Indeed, female players in our sample are on average younger than male players by about a year. However, the average career length of female players is not significantly different than that of male players (Guillaume et al., 2011). It is therefore unlikely that the differences in career life cycle can explain gender differences in the willingness to perform during the pandemic. We have added the discussion of this potential explanation to the “conclusions and discussion” section.

Guillaume M, et al. Success and decline: top 10 tennis players follow a biphasic course. Med Sci Sports Exerc. 2011 Nov 43(11):2148–54.
